# What Is the Carcass-Usage Mode of the Collembola? A Case Study of *Entomobrya proxima* in the Laboratory

**DOI:** 10.3390/insects10030067

**Published:** 2019-03-07

**Authors:** Lichao Feng, Liang Chang, Shaoqing Zhang, Xinyu Zhu, Sina Adl, Donghui Wu

**Affiliations:** 1 College of Earth Sciences, Jilin University, Changchun 130012, China; fenglichao@neigae.ac.cn; 2 Department of Plant Sciences, Jilin Agricultural Science and Technology College, Jilin 132101, China; 3 Northeast Institute of Geography and Agroecology, Chinese Academy of Sciences, Changchun 130102, China; springtail@neigae.ac.cn (L.C.); zhangshaoqing@neigae.ac.cn (S.Z.); 4 College of Resource and Environmental Sciences, Hebei Normal University, Shijiazhuang 050024, China; tia20021201@163.com; 5 Department of Soil Science, College of Agricultural and Bioresources, University of Saskatchewan, 51 Campus Drive, Saskatoon, SK S7N5A8, Canada; 6 Jilin Provincial Key Laboratory of Animal Resource Conservation and Utilization, Northeast Normal University, Changchun 130117, China; 7 Key Laboratory of Vegetation Ecology, Ministry of Education, Northeast Normal University, Changchun 130024, China

**Keywords:** food preference, invertebrate, microorganisms, food spoilage

## Abstract

Collembola display a variety of feeding habits, and prey on many types of food at different trophic levels in the soil. In most cases, their feeding selections are widely varied. In the interest of the food preferences of *E. proxima*, we attempted to confirm how the Collembola utilize food when feeding on carrion (unusual sources). Four different soil animals (with different stable isotope values and increasing trophic levels) were used to examine whether collembolans can use dead insects as a food resource in specific manners, depending on food preference. Our results demonstrated that the food preference of a collembolan changed significantly after feeding on insects with different feeding habits for 60 days. We found that stable isotope values (δ^13^C) of *Entomobrya proxima* approached those of the food sources. A large proportion of the diet (more than 50%) should directly consist of insect body parts, with the remainder consisting of indirectly used, mixed microorganisms naturally growing on animal food, such as fungi (*Rhizopus* sp., *Alternaria* sp., *Penicillium* sp., and *Aspergillus* sp.) and bacteria (*Bacillus* sp1. and *Bacillus* sp2.). Based on this research, the food preference of collembolans is more focused on carcasses (dead insect bodies) than microorganisms during the animal-food decomposition process.

## 1. Introduction

Collembola are among the most important decomposers [1,2,3,4], promoting the conversion of organic matter into inorganic matter in order to obtain high-quality carbon and nitrogen resources for growth, development and reproduction [5]. The feeding habits of Collembola are highly multifarious and include feeding on plant litter, fungi, bacteria, algae, live plant tissues, some plant pathogens, Protozoa, Nematoda, Rotatoria, Enchytraeidae and, carcasses [6,7,8,9,10,11]; their feeding habits are limited by food availability, abundance, quality and composition to some extent [12,13]. Microorganisms choose invertebrate food sources based on accessibility, digestibility and utilization [14,15]. In nature, dead organic matter (plant and animal residues) can be decomposed by microorganisms (fungi and bacteria) and invertebrates (for example dung beetles, march flies, maggots, mites, collembolans, and earthworms) in the soil [16,17,18], but the decomposition process of most parts is first carried out by microorganisms [19]. Thus, collembolans may feed on the food itself, the microorganisms, or both. As fungal feeders, collembolans are in contact with a large variety of fungi in their environment and experience a seasonal variation in the fungi [20]. The features of the temporal and spatial changes among fungal communities are closely connected with the high flexibility of epigeic Collembola in terms of conditions [16,21].

Thus, food preference is closely related to the range of vertical soil fauna activity [22], and this preference may be attributed to the restriction of feeding preference on utilization [23,24]. In general, detritivores obtain more nutrients or energy when consuming food at higher trophic levels [15], and they must rely on foods of different composition within an identical and environmentally homogeneous space [25]. Variability in the diet is an indication of complex trophic interactions, and members of Collembola benefit by feeding on polytrophic and abundant foods. Food resource availability is dynamic within a habitat, and collembolans thus encounter different foods in time and space. Because of such changes in food resources, the feeding habits and behaviors of collembolans vary with the composition, availability and palatability of a diet [13,26,27]. These habits and behaviors can be seen in fungal diet selection with fungal succession during the litter decaying process [28]. Overall, stable isotopes (δ^13^C) were used to detect changes in the diets of Collembola, because δ^13^C values differ largely among animals and life stages [29,30], and the δ^13^C and δ^15^N values of soil invertebrates vary over a wide range [31,32]. Moreover, each animal and stage has a unique stable isotopic ratio [33,34,35]. Therefore, δ^13^C is used to determine food selected in the process of food decomposition [36].

Collembola mouthparts, morphology and ecology. The mouth contain the labrum, a pair of mandibulae, a pair of maxillae, the hypopharynx and the bipartite labium. The frontal labrum, the ventral labium and two lateral oral folds enclose the other mouthparts in the buccal cavity [37]. Thus, Collembola can feed detritus and microorganisms [12]. Collembola inhabit soil, leaf litter, moss, the area under stones, caves, ant nests, etc., which are areas with wet and damp surroundings. They are the primary soil animals, and in forest soils, they can reach densities of 200 to 1800 ind. per dm^3^. In general, densities are only lower than the soil mites population [37,38]. Their living temperature is −4 °C to 28 °C, and relative humidity is 93% to 100% [39].

In this study, we used insect food to examine the food preference of *Entomobrya proxima* (an epigeic collembolan) in the spoilage process. We aimed to test whether the collembolan food-use mode is more complex and includes different utilization modes (direct intake or indirect use by microorganism).

## 2. Materials and Methods

### 2.1. Insect Food and Collembolan Activity

We selected the following soil invertebrates (insects) as artificial food with different feeding habits and trophic levels from habitats identical to those of *E. proxima* as food resources: Adults of an omnivorous ant (*Formica yessensis* Forel), a phytophagous scarab (*Popillia quadriguttata* Fabr), a saprophagous dung beetle (*Onthophagus viduus* Harold), and a carnivorous carabid (*Anoplogenius cyanescens* (Hope)) in a coniferous and broad-leaved mixed forest, located at 43.88° N, 126.57° E, Jilin, China. These insects were starved for 24 h [35] and killed by physical methods; the abdomens were removed and dried at 60 °C for more than 48 h until no further change in weight was observed. The residues were ground using a sterile mortar, and 30 mg was added to the substrate. Because of the presence of saprophytic microorganisms in the air or on the insect themselves, we wanted to check which saprophytic microorganisms would grow on the four food resources, and whether these microorganisms were foraged by the collembolan. First, we used PDA (potato dextrose agar) (Merck Co., Ltd., Kenilworth, NJ, USA) for fungi and NA (nutrient agar) (Merck Co., Ltd.) for bacteria in the air to identify which would grow on the animal food in our tests. Second, after some days of feeding, we inoculated the four insect powders that had been placed on the gypsum-activated carbon substrate onto PDA and NA to check for microorganisms on the insects. Fungi and bacteria were detected by using morphological and physicochemical characteristics and nucleotide sequences [40,41,42,43]. 

*E. proxima* was obtained using a suction trap. For rearing *E. proxima*, food was supplied as dried yeast (*Saccharomyces cerevisiae*; Angel Yeast Co., Ltd., Yi Chang, China) on a gypsum-activated carbon substrate in a plastic preservation box with a volume of 400 mL (plaster of Paris: activated charcoal at 9:1; Sinopharm Chemical Reagent Co., Ltd., Shanghai, China). Deionized distilled water was applied and maintained at temperatures under 17–19 °C. 

### 2.2. Feeding Strategies (E. proxima)

After seven days of starvation, 20 second-instar *E. proxima* in the second generation, with identical body sizes and levels of activity (10 days old), were inoculated onto each culture substrate [44] and cultured at 18 ± 1 °C and 85 ± 1% relative humidity with a light:dark cycle of 16:8 h (400–800 Lux) [45,46] while allowing for reproduction. Each day, deionized distilled water was added to maintain constant humidity. 

### 2.3. Stable Isotope Analysis

Isotopes were used to detect the *E. proxima* diet after 60 days. Although collembolans do not always consume a uniform amount of food in different instars or stages, the diet can be determined by the isotope composition of the consumer [47]. We used a stable isotope (*δ*^13^C) analysis to determine food preference and to verify food use. Based on the life cycle and development time, the life expectancy of *E. proxima* was more than 30 days under normal conditions (22–24 °C). The stable isotope (*δ*^13^C) analysis was conducted under a condition of sufficient food. We ground the tissues of the previously dried four soil fauna species to powders, which were then placed in tin capsules for elemental and stable isotope analyses. Fungi were also tested [48]. Samples were analyzed using a continuous-flow isotope ratio mass spectrometer (ThermoScientific Delta V Advantage; Waltham, Middlesex County, Massachusetts, USA). The *δ*-value is expressed as *δ*M(‰) = [(R_sample_/R_standard_) − 1] × 1000‰, where M is the element, ^13^C, R_sample_ is the^13^C/^12^C ratio of the measured samples, and R_standard_ represents the ratio of the heavy to the light isotope in an international standard [49].

### 2.4. Statistical Analysis

According to the mass conservation of stable isotopes, the number of possible food source combinations and acceptance solutions of food-source proportional contributions to a mixture based on stable isotope analyses were calculated by the IsoSource mixing model (https://isosource.software.informer.com/). This model provided the distribution of the source proportions that are consistent with isotopic mass balance [50,51]. The contribution ratio of different food sources after feeding by *E. proxima* was analyzed using a nonparametric Kruskal-Wallis test with a median test. The significance level of the statistical tests was *p* = 0.05. The statistical analyses were performed using R statistical software (‘ggpubr’, ‘digest’ packages) [52].

## 3. Results

**Figure 1:**
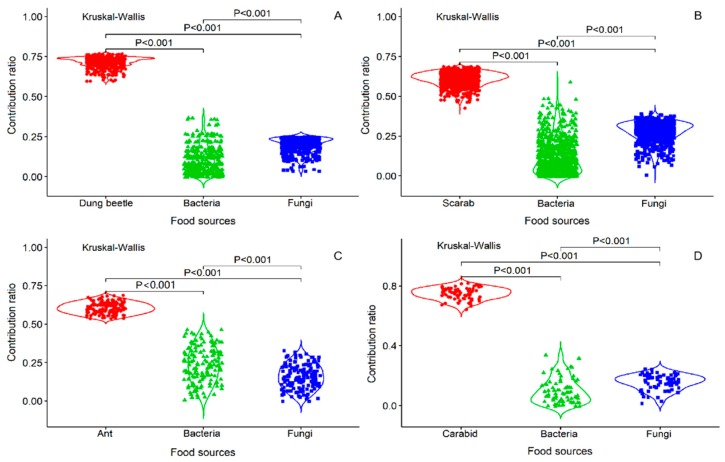
The bodies of four insects and microorganisms (fungi and bacteria) growing on insect bodies are the three types of food sources. Comparing each of the weighted averages of the number of combinations with the *δ_m_*, the comparative values for which the mass balance tolerances are between −0.1‰ and 0.1‰ are retained. For the contribution ratio in the figure, each dot represents the comparative values of the acceptable solution for the feeding proportion by *E. proxima*. Kruskal-Wallis explanatory values are statistically significant at *p* < 0.001.

**Figure 2:**
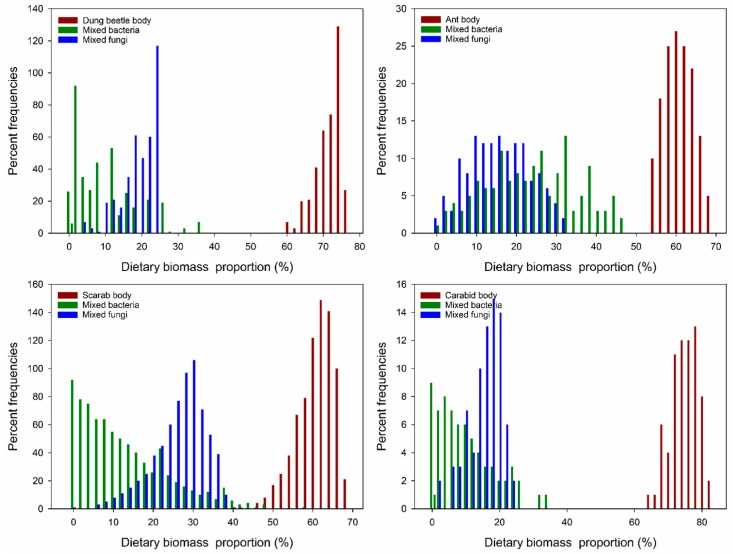
Dietary biomass proportions of the three aggregate food sources for *E. proxima*. The aggregate solutions are sums of the biomass contributions for the food sources in each category (insects body, mixed bacteria, and mixed fungi). Increments of 2% were used to calculate the aggregate contributions in the model iterations.

Four fungi (*Rhizopus* sp., *Alternaria* sp., *Penicillium* sp., and *Aspergillus* sp.) and two bacteria (*Bacillus* sp1. and *Bacillus* sp2.) grew on the insect body pieces during the decomposition process. The results of the analysis are listed in Table 1 as the data for calculating the acceptable solutions of food source contributions. By analyzing the food sources and Collembola after feeding, carcasses (insect body: Dung beetle, ant, scarab, and carabid) accounted for the large parts of food and ranged from 60–76%, 54–68%, 42–68%, and 64–82%, respectively. The isotopic values verified that the ratio of the four insect foods was higher than the ratios of the fungi and bacteria, especially for the carabids (*p* < 0.001, Figure 1A–D). In addition, by comparing the Collembola consumption ratios of the fungi and bacteria, more fungi than bacteria were consumed (*p* < 0.001, Figure 1A,B,D), except for the fungi growing on ants (*p* < 0.001, Figure 1C). These results indicate that *E. proxima* prefers to consume an insect body over microorganisms growing on an insect. The order of food selection was insect body, followed by fungi and then bacteria. For the mean percent frequencies of dietary biomass proportions, most of the carcasses accounted for a higher proportion than the microorganism carcasses, except for carabids. For the microorganism diet, Collembola consumed fungi more frequently (Figure 2).

## 4. Discussion

**Figure 3:**
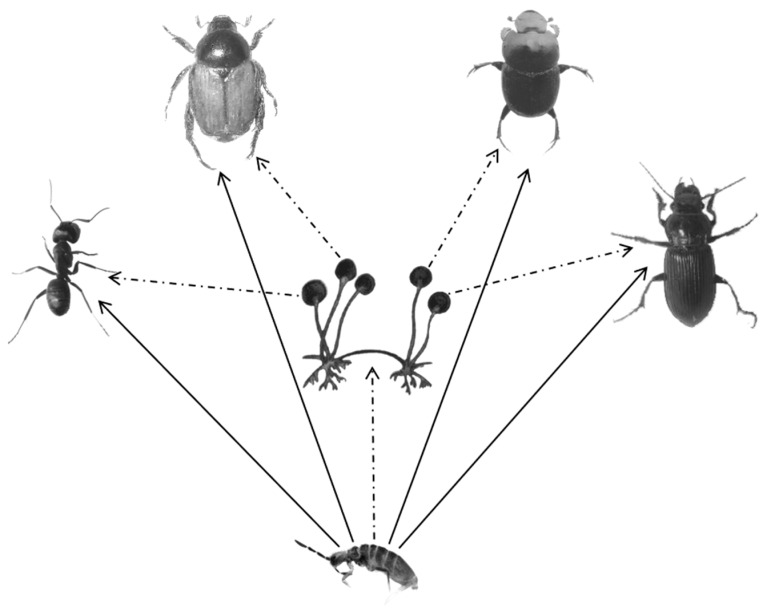
Each of the insects were a dominant soil organism in the same habitat as an epigeic collembolan. The solid line with an arrow indicates that the collembolan can directly digest the four types of insects, and the dotted line with an arrow indicates that the collembolan may consume microorganisms (such as fungi (*Rhizopus* sp., *Alternaria* sp., *Penicillium* sp., and *Aspergillus* sp.) and bacteria (*Bacillus* sp1. and *Bacillus* sp2.) in an indirect manner.

Based on our results, all food types resulted in different stable isotope ratios in the collembolan. Insect carrion was used more frequently than the microorganism by *E. proxima* during insect carcass decomposition. Direct exploitation (feeding carrion) would be more popular than indirect exploitation (Figure 3). These changes were determined by the eating patterns, and were also dependent on the food resource. For decomposition, microorganisms are typically the pioneer decomposers that decompose dead animals into carrion [19]. Carbon source assimilation explains the stable isotope value changes in the decomposer [53]. For decomposers, different assimilation intensities lead to different utilization and isotope values (*δ*^13^C) [54]. In natural habitats, it is difficult or impossible to verify the food source combination, because many food sources are used by the feeders, and the isotope signatures could be the same after being assimilated [51,54]. However, in our test, the aggregated food resources were determined, the dietary contributions were clearly displayed, and the IsoSource model can be applied to detect Collembola feeding habits. As fungal feeders, Collembola choose fungi as food [20,55], which is attributed to the nutrient content available in the habitat [12,56,57]. Although only 9% of animal residues have been found in the gut of most studied Collembola species and the other large parts were plant residues and fungi [58], after pre-decomposition by released microorganism enzymes, the carcasses would be more digestible for the decomposers [59,60]. 

## 5. Conclusions

We concluded that as decomposers, Collembola food utilization might be performed in many ways depending on the food sources available. In our study, for carcasses, Collembola were more focused on carrion (dead insect bodies) than microorganisms during the decomposition process.

## Figures and Tables

**Table 1:** Calculations of the stable isotope values and aggregate distributions of each food source to *E. proxima* after feeding on the insect body, fungi, and bacteria using the IsoSource model.

Insect Carcasses	Food Sources	Stable Isotope Value (*δ*^13^C) after Feeding by *E. proxima* (‰)	*δ_m_* Value	Median Value of of Each Dietary Aggregate Distribution (%)	Mean Percent Frequencies of Each Dietary Aggregate Distribution
Dung beetle	Body	−22.0530	−19.9473	72	43
	Mixed fungi	−13.5415		20	35
	Mixed bacteria	−17.1413		8	26
Scarab	Body	−24.7320	−20.2697	62	59
	Mixed Fungi	−11.6806		28	36
	Mixed bacteria	−17.0270		10	31
Ant	Body	−26.3250	−21.9593	60	18
	Mixed fungi	−12.6193		16	8
	Mixed bacteria	−17.0197		24	6
Carabid	Body	−20.7560	−19.2826	75	7
	Mixed fungi	−13.4896		17	7
	Mixed bacteria	−16.7463		8	5
